# Supplementary material for: The interactome of intact mitochondria by cross-linking mass spectrometry provides evidence for coexisting respiratory supercomplexes
Source: Mol Cell Proteomics. 2017 Dec 8;17(2):216–32. doi: 10.1074/mcp.RA117.000470 (PMC5795388; doi:10.1074/mcp.RA117.000470)
Supplement: Supplemental Data [file supp_RA117.000470_133922_0_supp_23978_fzffwf.zip › spectra_annotation/mito_spectra_annotation/441-1-14-1-19-1.pdf]

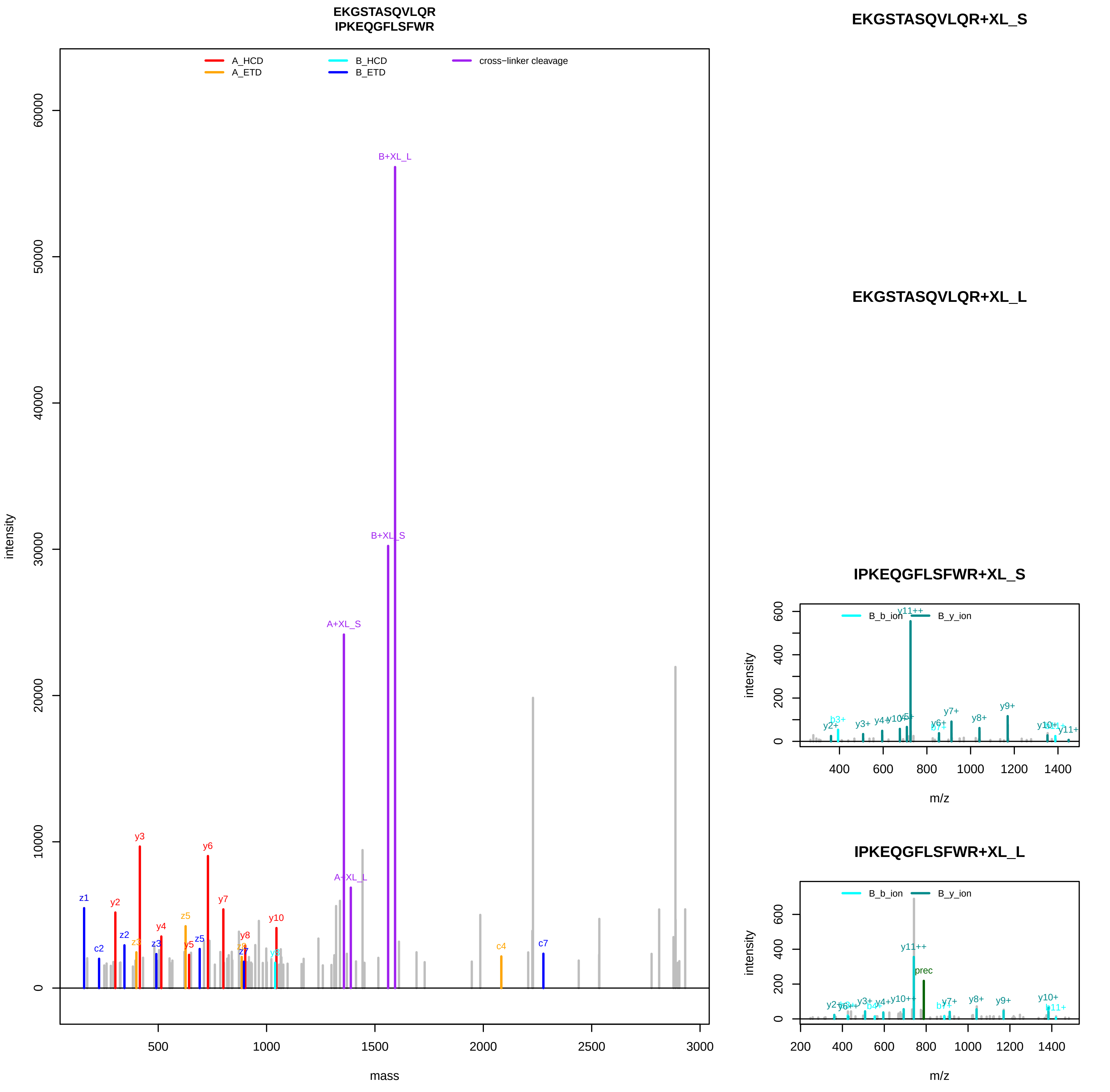
EKGSTASQVLQR
IPKEQGFLSFWR
A_HCD
A_ETD
B_HCD
B_ETD
cross−linker cleavage
B+XL_L
B+XL_S
A+XL_S
A+XL_L
z1
c2
y2
z2
z3
y3
z3
y4
z5
y5
z5
y6
y7
z6
y8
z7
y9
y10
c4
c7
0
10000
20000
30000
40000
50000
60000
500
1000
1500
2000
2500
3000
intensity
mass
EKGSTASQVLQR+XL_S
EKGSTASQVLQR+XL_L
IPKEQGFLSFWR+XL_S
B_b_ion
B_y_ion
y11++
y2+
b3+
y3+
y4+
y10++
y5+
y6+
b7+
y7+
y8+
y9+
y10+
b11+
y11+
0
200
400
600
400
600
800
1000
1200
1400
intensity
m/z
IPKEQGFLSFWR+XL_L
B_b_ion
B_y_ion
y11++
prec
y2+
y6++
y3+
b4+
y4+
y10++
b7+
y7+
y8+
y9+
y10+
b11+
0
200
400
600
200
400
600
800
1000
1200
1400
intensity
m/z
